# Complex reconstruction of the orbitofrontal regions using three regional flaps after orbital exenteration for the treatment of basal cell carcinoma

**Published:** 2020

**Authors:** Cătălin Gheorghe Bejinariu, Christiana Diana Maria Dragosloveanu, Silviu Adrian Marinescu

**Affiliations:** * Department of Plastic and Reconstructive Surgery, “Bagdasar-Arseni” Clinical Emergency Hospital, Bucharest, Romania; ** Clinical Ophthalmology Emergency Hospital, Bucharest, Romania

**Keywords:** orbital exenteration, giant basal cell carcinoma, facial reconstruction

## Abstract

**Objective.** The current paper presents an interesting case of facial reconstruction after the excision of a giant basal cell carcinoma located in the orbitofrontal region.

**Methods.** Performing the excision while securing the appropriate oncologic safety margin has determined the appearance of a soft tissue defect that required a complex reconstruction using three regional flaps: frontal, temporal fascial and temporal muscle flaps.

**Results.** After the excision and reconstruction in a single surgical stage, the postoperative result was favorable, the 12 months assessment showing that the patient was satisfied with the aesthetic aspect.

**Conclusion.** Including the orbital exenterations in the excisional treatment of giant neoplasms located in the facial region requires a complex reconstructive plan. The surgical team has to consider the relief of the anatomical structures that are targeted, as well as the necessity of achieving satisfactory aesthetic results while ensuring oncological radicality.

## Introduction

The excision of tumors located in the facial region requires the entire plastic surgeon’s armamentarium, considering the margins of oncological safety and subsequently reconstructing the anatomical regions affected by the neoplastic process. Regarding the cephalic region, the patients’ degree of tolerance related to the aesthetic result is very low compared to tumor pathology with another location.

The treatment of patients with giant tumors located in the orbitofrontal region should be performed by multidisciplinary teams that include, besides the plastic surgeon, the ophthalmologist, neurosurgeon, pathologist, and oncologist. The necessity of performing the orbital exenteration greatly increases the difficulty of the surgical intervention, contributing equally to the increase of the overall time of the surgery, as well as of the rate of complications associated with it [**1**]. The close collaboration between the plastic surgeon and the ophthalmologist during the entire duration of the treatment is essential in order to obtain good and stable results over time [**2**].

## Materials and methods – Case Report

The paper presents the case of a 65-year-old patient admitted to the Department of Plastic and Reconstructive Surgery of “Bagdasar–Arseni” Clinical Emergency Hospital for the treatment of a giant tumor located in the right orbital-frontal region. At the time of admission, the patient was undergoing treatment for high blood pressure and type II diabetes. 

**Fig. 1:**
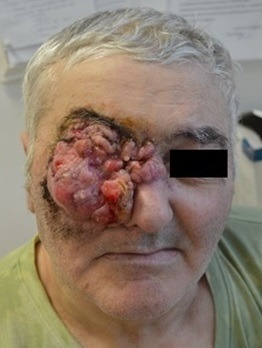
Preoperative aspect

The local examination revealed the presence of a spherical tumor with an irregular outline and a diameter of about 15 cm. The tumor was adherent to the neighboring structures, invading the right eyeball, dorsum nasi and the right zygomatic region (**Fig. 1**).

**Fig. 2:**
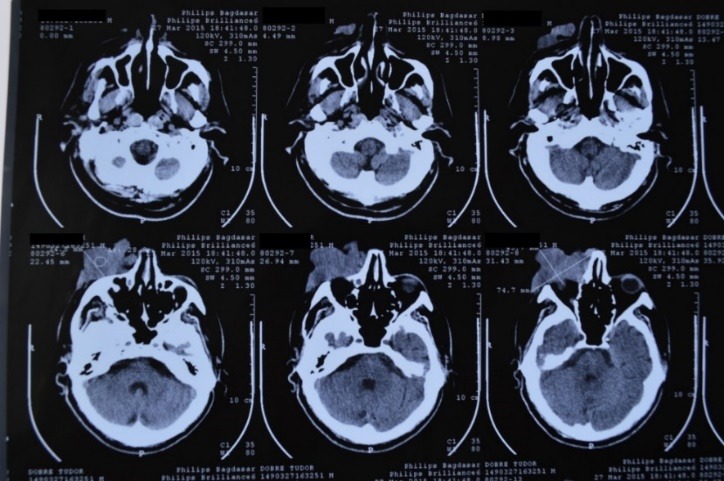
Anatomic relations of the tumor with the ophthalmic, nasal, maxillary, and zygomatic regions (computed tomography examination)

The computed tomography identified the presence of a space replacement process in the right zygomatic region, the ophthalmic region with posterior invasion of the right eyeball (**Fig. 2**).

**Fig. 3:**
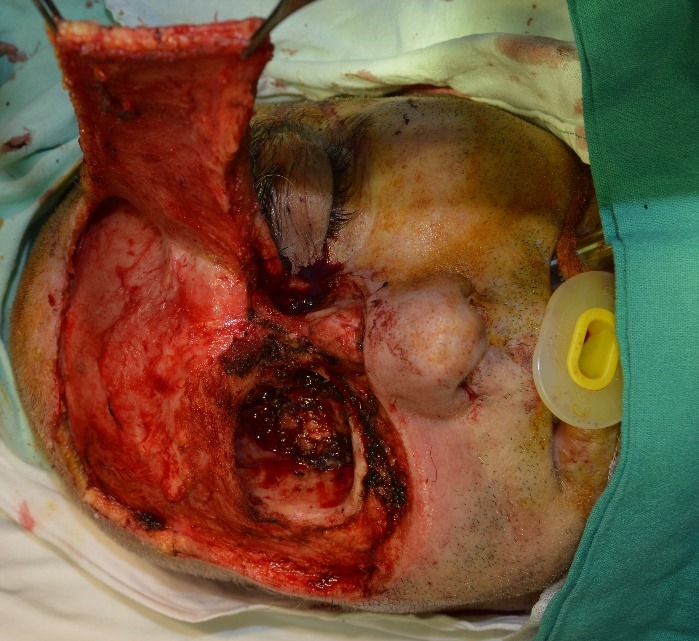
Intraoperative aspect after the excision and dissection of the frontal flap

The excision of the tumor was performed considering the margins of oncological safety, the excision piece being sent for intraoperative histopathological examination. After performing the excision, the reconstructive plan was structured to cover the defects by neighboring flaps, the pathological history of the patient making it impossible to perform a reconstruction using a free tissue transfer flap (**Fig. 3**).

**Fig. 4:**
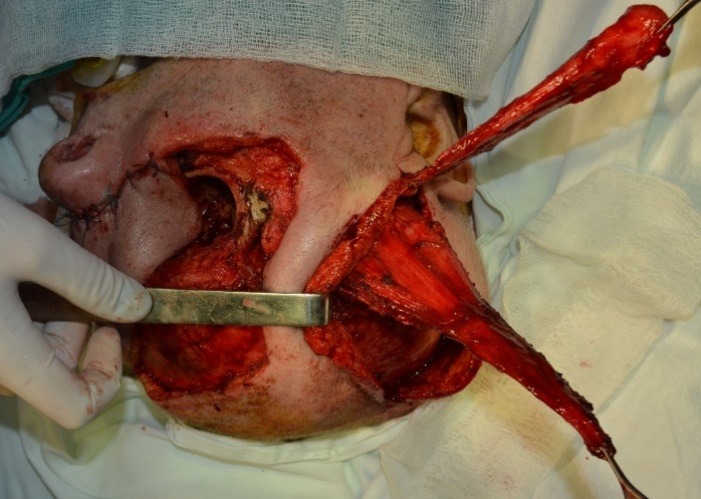
Intraoperative aspect after the dissection of the temporal flaps (fascial and muscular), including the frontal flap in the nasal region

A frontal flap was used to cover the dorsum nasi. The upper orbital region was reconstructed by rotating a temporal muscle flap, following that the orbital floor and the zygomatic region have benefitted from reconstruction using a temporal fascial flap (**Fig. 4**).

**Fig. 5:**
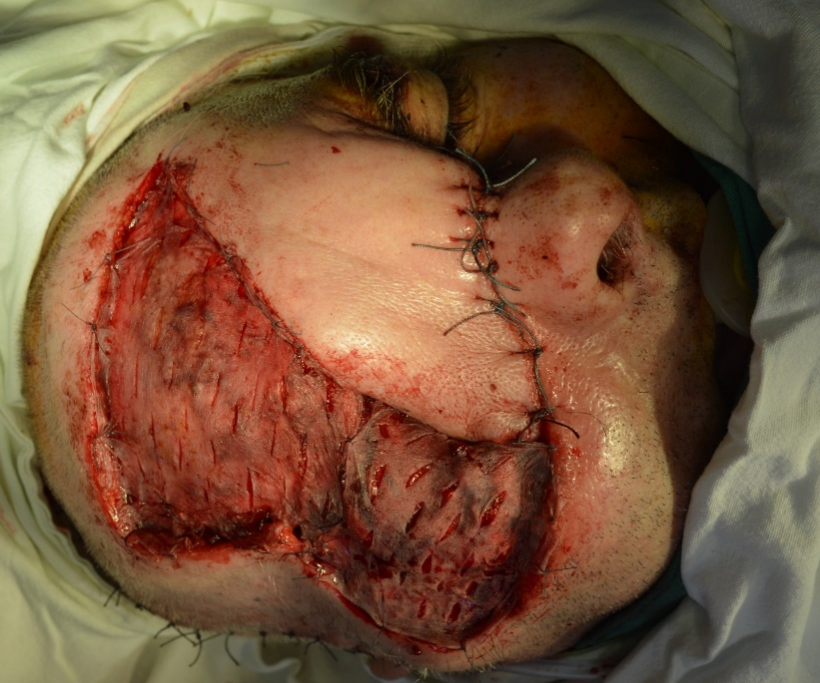
Intraoperative aspect at the end of reconstructive surgery

Surgery lasted 3 hours and was performed under general anesthesia, the patient being discharged 14 days after the surgical treatment (**Fig. 5**). Postoperative consults were performed regularly at every 3 months for 2 years.

## Results

As a result of the reconstruction, all 3 flaps had a favorable local evolution, the aesthetic aspect progressively improving according to the evaluations carried out at 3, 6 and 9 months after the intervention. The postoperative evolution was not marked by complications or other adverse events. 

**Fig. 6:**
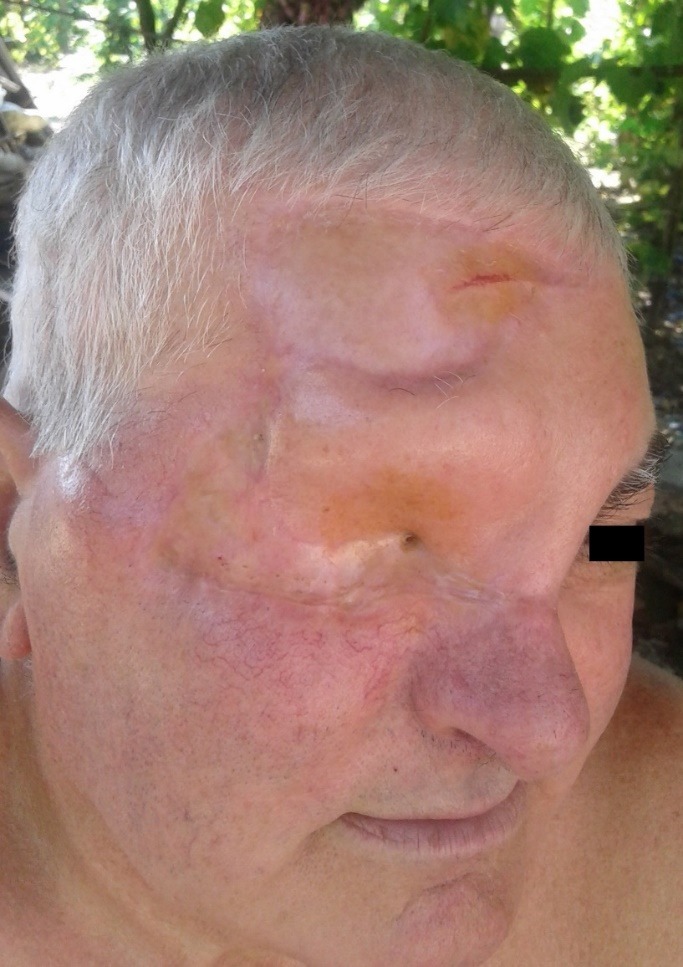
Postoperative aspect at 24 months after the surgery

Due to the severity of the orbital invasion by periocular basal cell carcinoma, it was not possible to preserve the visual function, the patient being able to successfully adapt to monocular vision (**Fig. 6**).

## Discussions

The treatment of facial neoplasms extended in the intra-orbital structures is extremely complex, being tolerated by patients with difficulty, in the conditions in which ensuring oncological radicality often requires orbital exenteration [**3**]. Performing the intraoperative histopathological examination is essential for the good course of the treatment, the complexity of the subsequent surgical interventions depending largely on the dimensions of the postexcisional defect [**4**]. In situations in which the patient’s pathological personal history is favorable, performing the reconstruction of large soft tissue defects using free tissue transfer flaps is the best solution.

The histopathological examination of the giant tumors located in the fronto-orbital region showed that the cell typology was associated with the accelerated growth rate of the neoplastic processes, also having a significant impact on the postoperative prognosis [**5**]. The histopathological analysis is a fundamental element during the therapeutic protocol of neoplastic processes, establishing the surgical indications, the profile of the therapeutic protocol and the postoperative behavior [**6**]. The international scientific literature dedicated to understanding the particularities and evolution of giant basal cell carcinomas showed that these tumors express neuroactive mediators, also presenting an accelerated rate of cell multiplication [**7**].

The frontal flap remains the main reconstructive option for soft tissue defects in the nasal region, especially in situations in which large radical excisions are performed [**8**].

The reconstruction of the upper orbital region is extremely difficult in cases in which total excision of the soft tissue is required, with large areas of bone exposure. These situations require the reconstruction based on muscle and fascia flaps, the rich vascular supply being an essential element in accelerating the healing and integration processes of skin grafts [**9**]. In order to achieve this goal, the temporal muscle flap has proven to be a very good solution, ensuring the premise of rapid healing, with stable results over time.

The reconstruction of the orbital floor was performed with a temporal fascial flap, thus creating the premises for a rapid integration of the skin graft. This type of flap has the advantage of great flexibility, having the particularity of maintaining its viability even under very high degrees of rotation [**10**]. The temporal fascial flap represents without a doubt a true “seat belt” in terms of reconstruction of the facial region in patients with giant tumors.

Knowing in detail the remaining reconstructive resources following the excision is essential to cover the soft tissue defects of large dimensions. Regardless of the anatomical region involved, reconstructive options need to be analyzed from a wide perspective [**11**], because not infrequently, the initial therapeutic protocol is changed intraoperatively due to the neoplastic extension to the neighboring structures.

The postoperative aesthetic aspect is an extremely important element that must be discussed in detail with the patient [**12**]. Giant tumors located at the level of the cephalic region are often associated with mutilating scarring [**13**]. The invasion of the orbital region by the neoplastic processes contributes in a significant manner to the outline of the mutilation nature of this pathology.

Performing the orbital exenterations to ensure the radicality of the oncological treatment is the main factor that contributes to the decrease of the patients’ satisfaction regarding the postoperative aesthetic result [**14**]. However, it should be mentioned that basal cell carcinoma is associated with an increased healing rate and consequently with a higher postoperative prognosis, compared to neoplastic pathology, such as squamocellular carcinoma or melanoma [**15**]. Regarding the presented case, the postoperative aesthetic result was considered satisfactory by the patient.

## Conclusions

Ensuring the radical excision of the tumor is the main component that influences the therapeutic algorithm.

Postexcisional reconstruction is more difficult as the size of the defect is larger. In these situations, the reconstructive protocol should include an overview of all available options and their possible associations.

Performing complex reconstructions involving the use of the temporal muscle flap associated with the temporal fascial flap and the frontal flap is possible and ensures good and stable results over time.

**Conflict of interest**

The authors declare no conflict of interest.

All authors agree with the publication of this manuscript.

This clinical investigation complies with the standards of the Ethics Committee of the “Bagdasar-Arseni” Clinical Emergency Hospital and adheres to the principles of the Declaration of Helsinki.

The patient was informed and signed the informed consent to participate in this study.
